# Quality of life impact of refractive correction (QIRC) results three years after SMILE and FS-LASIK

**DOI:** 10.1186/s12955-020-01362-8

**Published:** 2020-04-25

**Authors:** Tian Han, Ye Xu, Xiao Han, Jianmin Shang, Li Zeng, Xingtao Zhou

**Affiliations:** 1 The Key Lab of Myopia, Ministry of Health, Shanghai, People’s Republic of China; 2 Department of Ophthalmology and Vision Science, The Eye and ENT Hospital of Fudan University, No.19 Baoqing Road, Xuhui District, Shanghai, China; 3 Research Center of Ophthalmology and Optometry, Shanghai, China

**Keywords:** Long-term, SMILE, LASIK, Refractive surgery, Subjective, Glare, Dry eye symptom

## Abstract

**Background:**

This study aimed to compare long-term postoperative quality of life and satisfaction differences between SMILE and FS-LASIK for myopia correction.

**Methods:**

This cross-sectional study enrolled patients under the age of 39 years, who chose to undergo SMILE or FS-LASIK surgery to both eyes 3 years previously. Patients completed a common vision test and Quality of Life Impact of Refractive Correction (QIRC) questionnaire, together with the surgical satisfaction, adverse symptoms subjective survey. Patients with preoperative corrected distance visual acuity and postoperative uncorrected distance visual acuity of 20/20 or greater were included. Propensity score matching (PSM) was used to match the preoperative and postoperative spherical equivalent, age, and designed optical zones of the left and right eyes between the two groups.

**Results:**

Forty-nine patients were included in each group after PSM from 188 patients. No significant difference in the total QIRC score was found between the SMILE and FS-LASIK groups (45.89 ± 5.91 vs 45.09 ± 5.65, *p* = 0.492). There were no differences in surgical satisfaction between the groups (*p* = 0.178). Compared to the SMILE group, the FS-LASIK group had more glare (2.12 ± 2.25 vs 3.22 ± 2.54, *p* = 0.026) and severe dryness (1.80 ± 1.98 vs 2.79 ± 2.19, *p* = 0.021).

**Conclusion:**

Postoperative quality of life is similar after SMILE or FS-LASIK. Dry eye symptoms and glare were milder in the SMILE group than in the FS-LASIK group.

## Background

The ultimate goal of refractive surgeries is to improve the quality of vision and life. It is known that laser-assisted excimer laser in situ keratomileusis (LASIK) can lead to a dramatic improvement of quality of life [1–5]. Nowadays, femtosecond technology has brought new surgical methods: femtosecond laser-assisted LASIK (FS-LASIK), and femtosecond laser small incision lenticule extraction (SMILE). Of these, the SMILE procedure is relatively new and minimally invasive [6–9]. Both procedures are able to improve patients’ quality of life [10]. Ang et al. [7] compared 1-month and 3-month Quality of Life Impact of Refractive Correction (QIRC) questionnaire scores in patients who had undergone one of the two surgeries, and no statistically significant differences were found. However, Klokova et al. [11] found SMILE confered a better quality of life. To our knowledge, no study has yet compared long-term subjective scale scores of FS-LASIK and SMILE [12].

Many vision-related quality of life scales can be used to assess quality of life after refractive surgeries, including the QIRC questionnaire, National Eye Institute Visual Function Questionnaire, Refractive Status and Vision Profile, and Myopia-specific-Quality of Life Questionnaire. Most of above scales are based on classical test theory, QIRC uses both classical test theory and Rasch analysis, with great reliability and validity [10, 13]. The Rasch model estimates interval-level measurement on a continuous scale from ordinal items, and provides useful information for questionnaire development [10, 13].

The present study discusses the long-term QIRC outcomes, together with the satisfaction with surgery and adverse symptoms assessed by subjective survey after SMILE and FS-LASIK.

## Methods

### Participants

This is a retrospective cross-sectional study. Patients under the age of 39 years, who chose to undergo SMILE or FS-LASIK surgery at the Eye, Ear, Nose and Throat (EENT) Hospital of Fudan University in Shanghai, to both eyes 3 years previously, were asked to attend for a postoperative visit. Patients with a preoperative CDVA and postoperative UDVA of 20/20 or greater were studied. This study followed the tenets of the Declaration of Helsinki and was approved by the ethics committee of the EENT Hospital of Fudan University (KJ2008–10). Informed written consent was obtained from all participants.

### Surgery

The surgeries were all performed by the same surgeon (XZ). In the SMILE procedure, a 500 kHz VisuMax femtosecond laser system (Carl Zeiss Meditec, Jena, Germany) was used with pulse energy of 130 nJ. The lenticule diameter was set between 5.5 mm and 6.70 mm; the cap diameter was set to 7.5 mm at a 100 μm depth. A 90° single side cut with a length of 2.0 mm was created during the procedure. In the FS-LASIK group, the same femtosecond laser system was used for flap creation, followed by a Mel 80 excimer laser (Carl Zeiss Meditec) for stroma ablation, with a pulse energy of 185 nJ. The flaps had diameters of 8.5 mm and a thickness of 100 μm, with standard 90° hinges.

### Measurements

Patients were examined in terms of uncorrected distance visual acuity (UDVA), sphere, cylinder, and corrected distance visual acuity (CDVA). They also completed the QIRC questionnaire, together with the surgical satisfaction, adverse symptom subjective survey.

QIRC was developed by Pesudovs et al. [13], and adapted for use in Chinese by Xu et al. [14]. This scale includes a total of 20 items under the following four modules: postoperative symptoms, visual and physical functions, social activity, and mental health. It comprehensively evaluates postoperative changes in physical, physiological, psychological, and social health among the patients. Its Chinese edition has great reliability and validity, and can be used to clinically evaluate the quality of life in patients who have undergone refractive surgeries [10, 15].

This study employed the most commonly used survey of surgical satisfaction and adverse symptoms [16–19]. The surgical satisfaction survey contains two simple questions: “Are you satisfied with the procedure?”, and “Would you like to recommend the surgery to your friends and families?” Both questions were scored from 1 to 5, with complete satisfaction or willingness scored as 5, dissatisfaction or unwillingness scored as 1, and three integer scores in-between. The adverse symptoms survey scored 12 common postoperative adverse symptoms associated with corneal refractive surgery. All symptoms were scored from 0 to 10, with no feelings scored as 0, severe feelings scored as 10, and nine integer scores in-between.

### Statistical analysis

All statistical analyses were performed using the Statistical Package for Social Sciences (SPSS) (version 22; IBM, Armonk, NY, USA) and STATA 15.1 (StataCorp LP, College Station, TX, USA) software. The logistic regression approach without replacement was used in propensity score matching (PSM); the matching algorithm was the nearest neighbor with a match ratio of 1:1, and a caliper value of 0.02. PSM variables in the two groups included age, preoperative and postoperative spherical equivalent (SE), and designed optical zones of the left and right eyes. The main outcome measure was total score of QIRC. Thus, it was calculated that 34 patients in each group would achieve 90% power to detect a difference of 4, assuming a score of 45 with SD of 5 based on our previous study [15], with a significance level of 0.05. The independent t test and Mann-Whitney U test were performed. A *p* value of less than 0.05 was considered statistically significant.

## Results

Forty-nine patients were included in each group after PSM from 188 patients (Table 1). The outcomes of the QIRC questionnaire are shown in Table 2. No significant difference of the total QIRC score was found between the SMILE and FS-LASIK groups (45.89 ± 5.91 vs 45.09 ± 5.65, *p* = 0.492). The item with the lowest score in both groups was “How concerned are you about medical complications from your choice of optical correction?” in functional items (item 1–13).

**Table 1:** Characteristics of the SMILE and FS-LASIK Groups

	Entire cohort (***n*** = 188)					Propensity score-matched cohort (***n*** = 98)				
Characteristics	SMILE Group (***n*** = 97)		FS-LASIK Group (***n*** = 91)		P	SMILE Group (***n*** = 49)		FS-LASIK Group (***n*** = 49)		P
	Mean	SD	Mean	SD		Mean	SD	Mean	SD	
Age (years)	29.86	6.16	30.47	6.25	0.407	31.05	5.81	30.00	6.16	0.542
Preoperative SE (OD) (D)	−6.53	1.83	−7.99	2.52	< 0.001	− 6.99	1.95	−7.28	2.28	0.885
Preoperative SE (OS) (D)	−5.99	1.92	−8.15	2.50	< 0.001	−6.76	1.96	−6.97	2.53	0.939
Postoperative SE (OD) (D)	−0.22	0.56	− 0.51	0.96	0.024	−0.27	0.68	− 0.27	0.50	0.457
Postoperative SE (OS) (D)	−0.18	0.58	−0.53	1.05	0.205	−0.25	0.67	−0.23	0.53	0.555
Lenticule diameter (OD) (mm)	6.46	0.15	6.38	0.29	0.057	6.45	0.16	6.41	0.28	0.452
Lenticule diameter (OS) (mm)	6.46	0.15	6.38	0.29	0.104	6.45	0.16	6.42	0.27	0.696

*SMILE* small incision lenticule extraction, *FS-LASIK* femtosecond laser-assisted LASIK, *D* diopter

**Table 2:** Differences in QIRC Questionnaire Items between SMILE and FS-LASIK Groups

NO.	Questions	SMILE Group					FS-LASIK Group					Mean Ratio (SMILE/FS-LASIK)	P
		Mean	SD	Medium	Skewness	Kurtosis	Mean	SD	Medium	Skewness	Kurtosis		
Total score		45.89	5.91	45.94	0.18	−0.01	45.09	5.65	45.24	0.23	0.10	1.02	0.492
Total score (Q1 to Q13)		47.88	5.71	47.59	0.00	0.16	45.82	6.56	46.40	0.17	0.30	1.04	0.100
Total score (Q14 to Q20)		42.21	9.62	40.99	0.49	−0.34	43.73	9.03	45.36	−0.14	− 0.96	0.97	0.421
1	How much difficulty do you have driving in glare conditions?	46.32	10.38	45.06	−0.10	− 0.70	40.65	10.46	45.06	0.42	−0.75	1.14	0.009
2	During the past month, how often have you experienced your eyes feeling tired or strained?	46.82	7.51	49.66	−0.46	0.54	46.82	8.15	49.66	−0.20	0.24	1.00	0.968
3	How much trouble is not being able to use off-the-shelf (non prescription) sunglasses?	38.11	10.45	41.26	0.27	−0.76	37.79	12.32	41.26	0.43	−1.29	1.01	0.753
4	How much trouble is having to think about your spectacles or contact lenses or your eyes after refractive surgery before doing things; eg, traveling, sport, going swimming?	51.60	9.82	45.92	−0.49	−0.60	49.07	11.79	45.92	−0.37	−1.17	1.05	0.329
5	How much trouble is not being able to see when you wake up; eg, to go to the bathroom, look after a baby, see alarm clock?	47.97	10.35	43.87	−0.37	−0.73	45.45	11.90	43.87	−0.18	−1.27	1.06	0.306
6	How much trouble is not being able to see when you are on the beach or swimming in the sea or pool, because you do these activities without spectacles or contact lenses?	61.08	6.04	63.92	−1.69	0.88	57.62	9.41	63.92	−1.23	0.54	1.06	0.053
7	How much trouble is your spectacles or contact lenses when you wear them when using a gym/ doing keep-fit classes/circuit training, etc.?	49.81	8.67	55.17	−1.38	1.05	46.66	10.95	55.17	−0.91	−0.43	1.07	0.153
8	How concerned are you about the initial and ongoing cost to buy your current spectacles/contact lenses/refractive surgery?	57.36	10.97	64.61	−1.20	0.09	55.78	11.37	64.61	−0.89	−0.57	1.03	0.439
9	How concerned are you about the cost of unscheduled maintenance of your spectacles/ contact lenses/refractive surgery; eg, breakage, loss, new eye problems?	51.17	10.84	60.62	−0.71	−0.65	48.01	12.48	45.18	−0.35	−1.38	1.07	0.223
10	How concerned are you about having to increasingly rely on your spectacles or contact lenses since you started to wear them?	53.79	11.60	50.01	−0.44	−1.08	51.27	11.73	50.01	−0.14	−1.22	1.05	0.279
11	How concerned are you about your vision not being as good as it could be?	39.60	9.22	34.24	1.55	1.44	38.65	9.46	34.24	2.03	2.91	1.02	0.421
12	How concerned are you about medical complications from your choice of optical correction (spectacles, contact lenses and/or refractive surgery)?	37.10	10.49	28.59	0.85	−0.39	35.84	9.51	28.59	0.96	−0.04	1.04	0.593
13	How concerned are you about eye protection from ultraviolet (UV) radiation?	41.71	9.36	35.72	1.33	0.80	43.92	10.51	35.72	0.92	−0.30	0.95	0.277
14	During the past month, how much of the time have you felt that you have looked your best?	51.52	15.77	60.79	−0.27	− 0.89	47.20	17.94	45.52	0.26	−1.29	1.09	0.202
15	During the past month, how much of the time have you felt that you think others see you the way you would like them to (eg, intelligent, sophisticated, successful, cool, etc)?	44.08	12.03	48.99	0.35	−1.14	44.08	12.03	48.99	0.35	−1.14	1.00	1.000
16	During the past month, how much of the time have you felt complimented/flattered?	48.94	14.51	37.28	1.04	0.30	50.97	12.39	54.55	0.22	−1.26	0.96	0.277
17	During the past month, how much of the time have you felt confident?	42.91	14.37	42.67	0.52	−0.11	42.16	12.99	42.67	0.21	−0.53	1.02	0.921
18	During the past month, how much of the time have you felt happy?	40.10	14.04	39.61	0.29	−0.50	44.13	12.54	39.61	−0.03	−0.08	0.91	0.127
19	During the past month, how much of the time have you felt able to do the things you want to do?	28.16	13.89	31.66	0.79	0.23	32.73	14.73	31.66	0.15	−0.89	0.86	0.096
20	During the past month, how much of the time have you felt eager to try new things?	39.72	14.11	41.22	0.52	−0.28	44.83	15.37	41.22	0.33	−0.48	0.89	0.100

*QIRC* Quality of Life Impact of Refractive Correction

There were also no differences in surgical satisfaction and recommendations between the SMILE and FS-LASIK groups (4.39 ± 0.76 vs 4.16 ± 0.87, *p* = 0.178; 4.33 ± 0.75 vs 4.33 ± 0.72, *p* = 0.934).

**Fig. 1:**
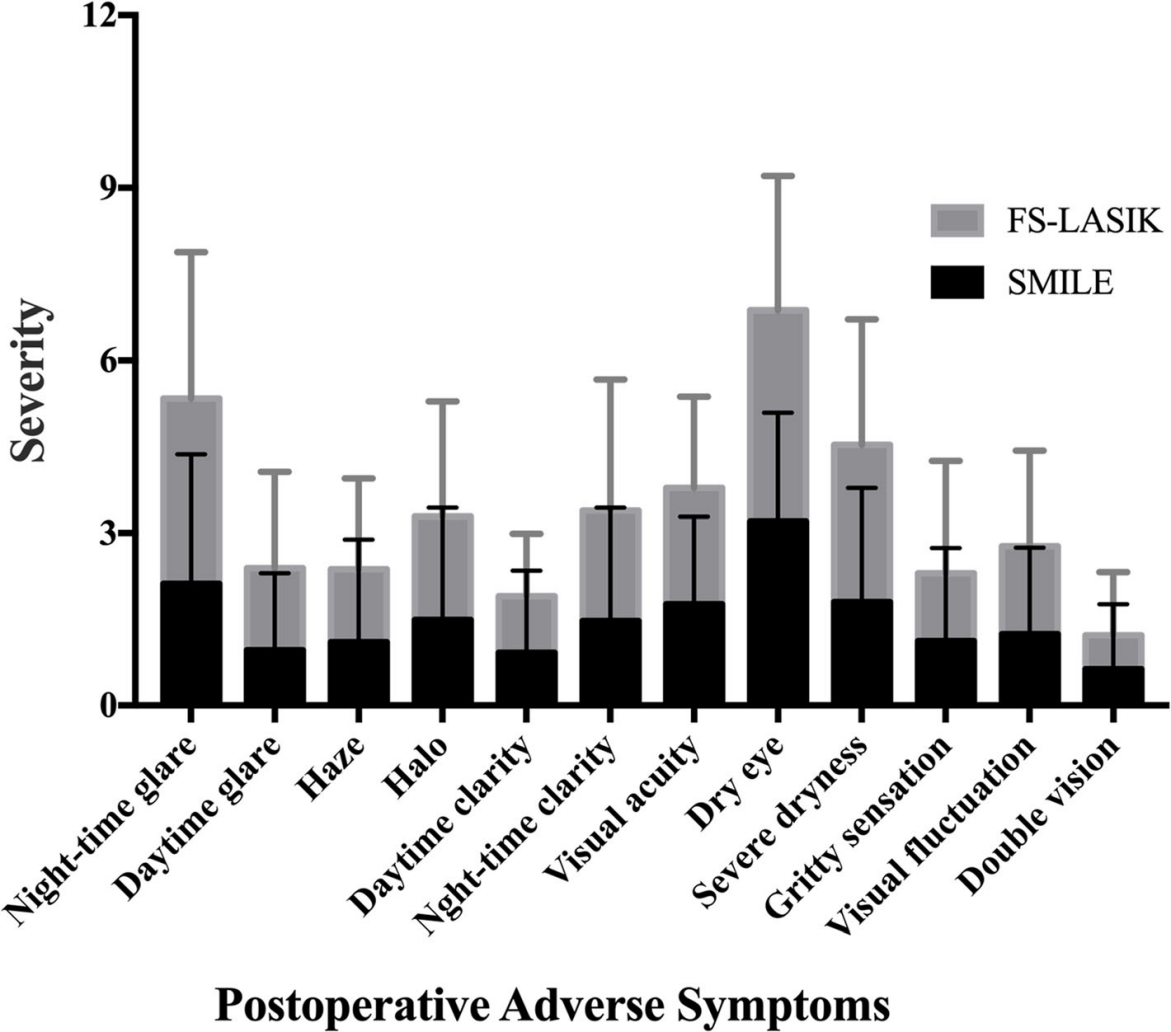
Postoperative adverse symptoms scores between SMILE and FS-LASIK

The outcomes for 12 symptoms are shown in Fig. 1. Compared to the SMILE group, the FS-LASIK group had more glare (2.12 ± 2.25 vs 3.22 ± 2.54, *p* = 0.026) and severe dryness (1.80 ± 1.98 vs 2.79 ± 2.19, *p* = 0.021). The total score was similar between the two group (17.82 ± 13.17 vs 22.35 ± 13.84, *p* = 0.065).

## Discussion

Subjective scales that assess postoperative satisfaction and quality of life with long-term follow-up are necessary to enhance the current understanding of the efficacy and adverse effects of the two surgical methods, and thus be beneficial to the development and widen the acceptance of corneal refractive surgeries. In the present study, we used postoperative subjective questionnaires for the long-term assessment of FS-LASIK and SMILE.

In order to present fair and objective results, the present study used PSM for the selection of subjects. In order to avoid variations caused by visual acuity and refractive power on the vision quality [20, 21], all of the subjects who were selected had preoperative CDVA and postoperative UDVA of at least 20/20, and the postoperative SE of the left and right eyes were matched separately between the two groups. Designed optical zones, which also have an influence on vision quality [8, 22], were also matched. Additionally, considering that lower satisfaction with corneal refractive surgery is found in older patients compared to younger patients [4], age is also one of PSM parameters.

The present study found similar results regarding surgical satisfaction to the short-term questionnaire survey study [7, 12], with no significant difference in the total QIRC score of the SMILE and FS-LASIK groups. Moreover, there were no between-group differences in the surgical satisfaction, degree of recommendation, total score of QIRC items 1–13, and total score of QIRC items 14–20. The total QIRC score in the SMILE group in our study (45.89 ± 5.91) was similar to our 4-year outcomes (45.71 ± 2.61) [15]. The total QIRC score in the FS-LASIK group in this study (45.09 ± 5.65) was smaller than that found in studies by Meidani et al. [10] (53.7 ± 5.1) and Garamendi et al. [1] (53.09 ± 5.25). This might due to the conservative nature of Chinese people.

In both groups, the QIRC item with the lowest score in functional items was “Are you concerned about complications from your current method of optical correction?”. This showed that patients were most concerned about the surgical safety, which is most important for the development of corneal refractive surgery. Doctors should pay attention to their preoperative discussions with patients in order to ease concerns [23].

Medical examinations of vision acuity, preoperative and postoperative SE, that are usually used to assess the efficiency of refractive surgeries, cannot equal to patients’ subjective assessment of vision recovery. Symptoms such as dry eye, glare, and halos may still bother patients, even with a UDVA of 20/20. Similar to other studies [6, 24], dry eye symptoms and glare were the main problems in both groups. Moreover, the SMILE group was found to have less severe dry eye-related results compared to the FS-LASIK group. Theoretically, dry eye symptoms are caused because corneal refractive surgery severs the corneal nerves, and reduces the secretion of tear-associated factors. In the FS-LASIK procedure, a 20-mm incision is made, with a flap-lifting step. In contrast, the incision in the SMILE procedure is only 2 mm, corneal nerve injury is reduced, and the postoperative symptoms of dry eye and decreased corneal sensation are alleviated [25–27]. This has also been proved in meta-analyses outcomes [28, 29].

In this study, the SMILE group had better glare-related outcomes when compared to the FS-LASIK group. Glare is partly caused at the transitional region between the ablated and non-ablated tissues in the pupil following corneal refractive surgery. Light rays inside the eye do not regularly scatter onto the retina but form a curtain of light. This leads to decreased contrast sensitivity of images formed on the retina [30, 31], and halos [32], and affects night driving. When the designed optical zone was matched, the actual optical zone of the SMILE procedure was found to be larger than that of the LASIK procedure [8, 22], which resulted in less irregular scattering of light and might have contributed to the results of the present study. Moreover, the maximum scores were 10, and the scores of the two groups were both approximately 0–3, showing that glare-related symptoms were not very serious. This finding accords with those of other studies, in that the majority of cases recovered from glare several mouths postoperatively [6, 33–35].

Limitations of the present study are that it was a cross-sectional study rather than a randomized clinical trial, and did not collect preoperative and other time point questionnaire results from the same patients. However, considering that the patients in the two groups were carefully matched, and that bias exists in both groups, some concerns could be mitigated.

## Conclusion

In summary, postoperative quality of life is similar after SMILE or FS-LASIK. Dry eye symptoms and glare were milder in the SMILE group when compared to the FS-LASIK group.

## Data Availability

Available upon request from the first author; Dr. Tian Han.

## Abbreviations

FS-LASIK: Femtosecond laser-assisted excimer laser in situ keratomileusis
SMILE: Femtosecond laser small incision lenticule extraction
SE: Spherical equivalent
QIRC: Quality of life impact of refractive correction
